# A canine model of experimental infection with *Leishmania (L.) mexicana*

**DOI:** 10.1186/1756-3305-7-361

**Published:** 2014-08-09

**Authors:** Julio Vladimir Cruz-Chan, Amarú del Carmen Aguilar-Cetina, Liliana Estefanía Villanueva-Lizama, Pedro Pablo Martínez-Vega, Maria Jesús Ramírez-Sierra, Miguel Enrique Rosado-Vallado, José Leonardo Guillermo-Cordero, Eric Dumonteil

**Affiliations:** Laboratorio de Parasitología, Centro de Investigaciones Regionales “Dr. Hideyo Noguchi”, Universidad Autónoma de Yucatán, Calle 96 S/N x Av. Jacinto Canek y Calle 47, 97225 Mérida, Yucatán Mexico; Department of Tropical Medicine, School of Public Health and Tropical Medicine, Tulane University, New Orleans, LA USA; Campus de Ciencias Biológicas y Agropecuarias, Universidad Autónoma de Yucatán, Mérida, Yucatán México

**Keywords:** Leishmaniasis, Canine model, Experimental infection

## Abstract

**Background:**

Cutaneous leishmaniasis is a tropical disease affecting over one million patients annually and *Leishmania (L.) mexicana* is one of the major etiological agents in the Americas. Here we established the first experimental infection of *L. (L.) mexicana* in canids.

**Methods:**

Beagle dogs were infected intradermally with culture-derived *L. (L.) mexicana*. We followed skin ulcer development, histopathological signs, parasite burden and the immune status of the infected dogs.

**Results:**

All infected dogs developed uniform oval-craterform ulcers similar to those observed in humans, associated with mixed T helper 1/T helper 2 immune responses. Parasites were detected in the healed lesions 15 weeks post-infection. Higher anti-*Leishmania* IgG levels correlated with larger lesions and high IgG1/IgG2 ratio was associated with some level of splenomegaly.

**Conclusions:**

The canine model described in this work will be of use for further understanding of *L. (L.) mexicana* immunopathogenensis, and for drug and vaccine development.

## Background

The leishmaniases are a group of parasitic disease affecting up to 1.4 millions people annually [1]. Cutaneous (Chiclero’s Ulcer), mucocutaneous and visceral leishmaniasis (Kala-azar) are the different clinical forms of the disease, which is caused by parasites from two subgenus: *Leishmania (Leishmania)* and *Leishmania (Viannia).* The parasite is transmitted by sand flies of the *Phlebotomus* and *Lutzomyia* genus in the old and new world, respectively. Cutaneous leishmaniasis (CL) ranks ninth of infectious neglected-tropical diseases, with an estimated burden of 1.2 million new cases per year, and of 770,000 DALYs (disability-adjusted life years) [1–3]. *L. (L.) mexicana* is one of the major species responsible for CL in the Americas. It can be found in Argentina, Brazil, Costa Rica, Guatemala, and as far north as Mexico [1, 4, 5]. In Mexico, the Ministry of Health reports an incidence of 500-900 cases annually, although the disease is likely to be under-reported [5, 6]. Drug treatment for infected patients is complicated, with only few drugs available with limited efficacy, and a vaccine is still in early experimental stages [1, 5, 7, 8].

Dogs are believed to play an important role as a domestic reservoir of *Leishmania* parasites, as demonstrated for *L. (L.) chagasi* [9]. This is due to relatively high natural infection rates in many regions, ranging from 4 to 60% [10–15] and the life-long persistence of parasites, particularly in the skin, with or without clinical signs of disease [9]. Drug treatment of infected dogs is not recommended, to prevent the emergence of drug resistance of the parasite [16]. As a consequence, some countries such as Brazil are recommending the culling of infected dogs to prevent spreading of the disease, although the efficacy of such measure has been criticized [17]. An important priority is thus to develop veterinary vaccines, which require appropriate animal models for their evaluation. Rodent models have been extensively used for the study of *Leishmania* infections, including inbred and outbred mouse strains and hamsters [18, 19], and have provided key information on the immunopathology of the disease. However, studies in dogs are also warranted for a further understanding of their role in *Leishmania* transmission and for testing drug and vaccine efficacy [20, 21].

Most studies on canine leishmaniasis have focused on the visceral form caused by *L. (L.) chagasi* or *L. (L.) infantum*, and are based on observations of both naturally and experimentally infected animals [21–27]. Unexpectedly, canine cutaneous leishmaniasis has been little studied in domestic dogs and is poorly described. A high prevalence of infection by *L. (V.) braziliensis*, *L. (V.) guayanensis*, and *L. (L.) panamensis* has been reported in dogs from Colombia [27, 28]. In Mexico, cases of canine leishmaniasis have been reported, possibly caused by *L. (L.) infantum* [29], although the parasite species was not determined. Cases of natural infection with *L. (L.) mexicana* have also been described [30]. Dogs naturally infected with *L. (V.) braziliensis* were found to present thrombocytopenia, anemia and skin lesions [31]. Parasites could be observed in 35% of the lesions, which also presented diffuse chronic inflammation of the dermis and fibrinoid degeneration, and less frequently some vasculitis [32]. However, experimental models of canine cutaneous leishmaniasis are scarce. The experimental infection with *L. (V.) brasiliensis* was found to induce a cutaneous lesion in three of four mongrel dogs, 4-8 months after infection, and the lesions tended to heal after 3-5 months [33]. Histologically, these lesions had similar characteristics as those observed in naturally infected animals [33], but additional studies are needed to further characterize this dog model.

In this study, we developed a canine model of cutaneous leishmaniasis caused by experimental infection with *L. (L.) mexicana* parasites, the main species circulating in Southern Mexico and Central America. We described the clinical, parasitological and immunological aspects of the experimental infection in Beagle dogs, which provide a good model for the future testing of novel drugs or vaccines against cutaneous leishmaniasis, as well as for further studies on the immunopathogenensis of this canine host.

## Methods

### Animals

Six Beagle dogs from three litters and aged between two and three months were used. Animals were acclimated for three/four months in the animal facility. Temperature, light and food were controlled. They received treatment against helminths and were vaccinated against Rabies virus, Canine distemper virus, Type 2 Adenovirus, Coronavirus, Parainfluenza, Parvovirus and Leptospira. All animal procedures were performed according to national and international guidelines approved by the Institutional Bioethics Committee from the Autonomous University of Yucatan (authorization number CBI-CIR-11-04).

### Parasites and infection

*Leishmania (L.) mexicana* MHET/MX/97/Hd18 strain was cultured in 199 media with 15% of fetal bovine serum, 5 μM mercaptoethanol, 20 mM sodium pyruvate, 100 IU penicillin and 100 mg/mL streptomycin, and 5% of filtered human urine. Under general anesthesia (ketamine/xilazine, 8:1 mg/kg, iv), Beagle dogs received 7 × 10^7^ promastigotes resuspended in 50 μl of PBS solution intradermally on the shaved back [34].

### Clinical studies

Beagle dogs were under daily examination for clinical signs including rectal temperature, breath and heart rate. Additionally, feces color, presence of polydipsia, polyuria and/or any abnormal excretions were observed. Skin lesions were photographed and measured weekly using a Vernier Caliper over a period of 15 weeks.

### Parasite burden

SYBR Green-based real time-PCR was optimized to quantify *L. (L.) mexicana* parasite burden in prescapular lymph nodes and skin lesions. First, 25 mg of tissue was ground with a morter and pestle and DNA was obtained with a Wizard® Genomic DNA purification Kit (Promega Madison WI) following the manufacturer’s instructions.

We used primers targeting a 140 bp sequence from *L. (L.) mexicana* minicircle: forward primer: 5′-AATGCGAGTGTTGCCCTTTTG-3′ and reverse primer: 5′-GCCGAACAACGCCATATTAACC-3′ [35]. Reactions contained 50 ng of DNA in a reaction volume of 20 μl with 500 nM (each) forward and reverse primers, and 1× Kappa SYBR FAST universal qPCR mix. Reactions consisted of a 10 min activation at 95°C followed by 40 cycles of 15 s at 95°C and 1 min 60°C, and a high resolution melt curve analysis at the end of the reaction. A standard curve was prepared with uninfected dog DNA spiked with serial dilutions of *L. (L.) mexicana* DNA covering a dynamic range of 150 to 1.5 × 10^6^ parasite equivalent/reaction. All samples and standards were run in triplicates and the standard deviation among triplicates was less than 0.6 Cq.

### Pathology

Dogs were euthanized 15 weeks post-infection with a barbiturate overdose and necropsies were performed immediately registering detail of cavities, organs and fluids. Spleen and Liver were extracted, weighted and measured [36]. Biopsies from the skin, spleen, prescapular lymph node and liver tissues were embedded in paraffin and sections were stained with hematoxylin and eosin to evaluate lesions and/or parasites.

### Serology

The humoral immune response induced by *L. (L.) mexicana* infection was evaluated by measuring levels of total IgG, IgG1 and IgG2 subtypes, against parasite lysate and NH36 recombinant antigen [37, 38]. Soluble *L. (L.) mexicana* Antigen (SLA) was prepared from parasites cultured as described previously following 6 days culture in 199 Hanks® medium (Life technologies Carlsbad CA). Briefly, 4 × 10^5^/mL parasites were washed three times with phosphate buffer solution (PBS) pH 7.2 and sonicated by three ultrasound cycles (Sonics Vibra Cell 130, Newtown CA) of 1 min at 45 W at 4°C. The lysed suspension was centrifuged at 5000 × g for 30 min at 4°C. The supernatant was recollected and stored in aliquots at 70°C until used [39]. Recombinant NH36 (rNH36) was generously provided by Dr. Peter Hotez (Baylor College of Medicine, Houston, TX, USA). Ninety six-well plates were coated overnight with soluble *L. (L.) mexicana* MHET/MX/97/Hd18 antigen (20 μg/ml) or rNH36 recombinant protein (2.5 μg/ml). After blocking, plasma samples (1:100 and 1:50 dilutions for total IgG and IgG isotypes, respectively) were incubated for 1 h at 37°C, and then washed. Phosphatase-conjugated rabbit anti-dog IgG (1:4000 dilution, Sigma, USA), peroxidase-conjugated goat anti-dog IgG1 or sheep anti-dog IgG2 (1:1000 dilution, Serotec, UK) were added and after washing, the plates were revealed with pNPP or o-phenylenediamine substrates, respectively, and absorbance was measured on a Bio-Rad 550 microplate reader.

### Cytokine levels

Cytokines IFNγ and IL-10 levels were quantified in plasma as well as spleen and liver extract supernatants. For this, 1 g of fresh tissue was ground with a mortar and pestle and resuspended with 2 mL of RPMI-1640 medium (Sigma-Aldrich Inc, St. Louis, MO). The homogenates were centrifuged at 10,000 *g* for 15 minutes at 4°C and the supernatants were stored at -70°C [40]. IFNγ and IL-10 DuoSet Canine kits (R&D Systems Inc., Minneapolis) were used to measure cytokine levels in tissue supernantants and plasma samples according to the instructions of the manufacturer.

## Results

### Clinical evaluation

**Figure 1:**
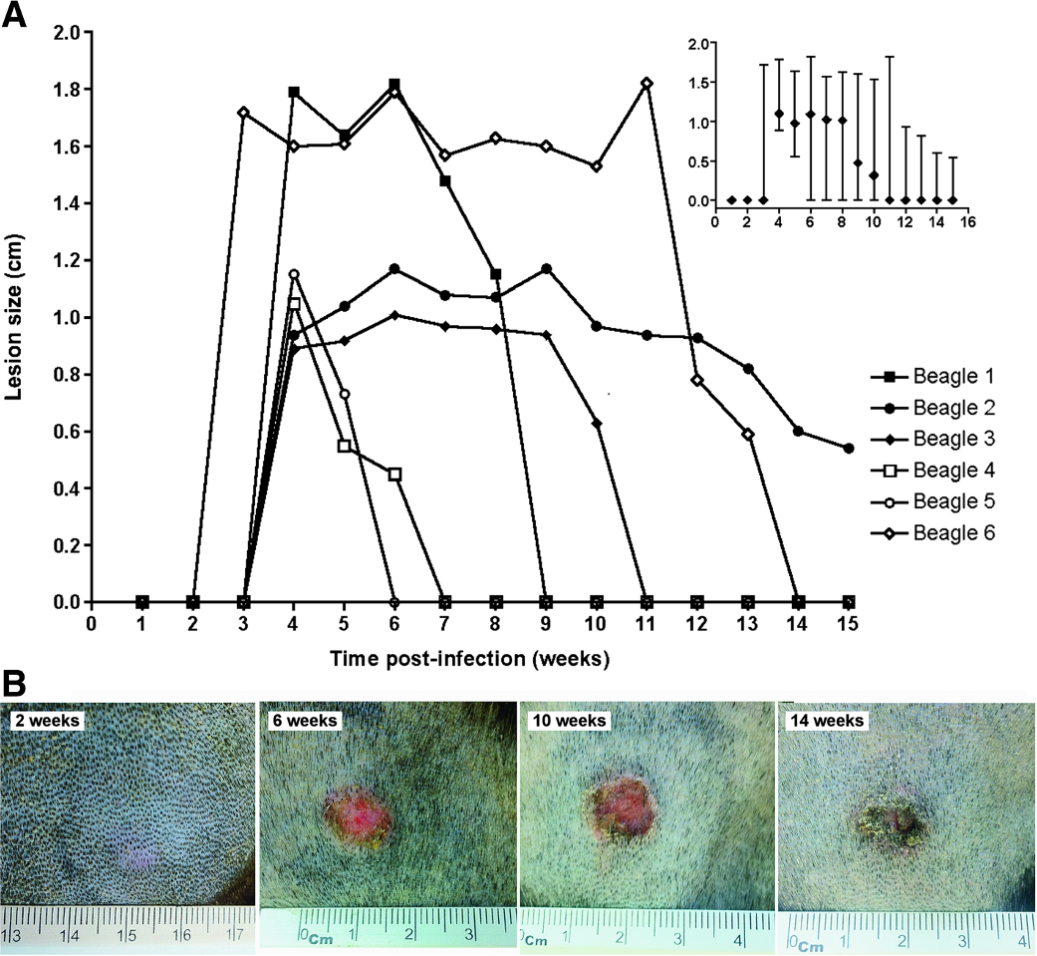
**Time course of skin lesion development in dogs infected with** ***L. (L.) mexicana*** **.** Beagle dogs were infected with 7 × 10^7^ parasites intradermally and lesion size was measured weekly with a caliper **(A)**. Black filled symbols are male dogs and empty symbols are for females. Insert shows the median and range of the lesion size. **(B)** representative images of lesions at 2, 6, 10 and 14 weeks post-infection. Scale is in cm.

Dogs were infected with a dose of 7 × 10^7^ *L. (L.) mexicana* promastigotes via intradermal route. All animals showed a local erythema nine days post-infection at the site of injection. At 3-4 weeks post-infection, we observed a papule in all animals, which ulcerated and expanded, to form a typical oval crater-like lesion (Figure 1). The majority of animals presented a lesion of about 1 cm in diameter, with some variation (range of 0.4 - 1.8 cm in diameter). Active skin ulcers were present for a median of 8 weeks until healing, with large individual variations of this duration, ranging from six to 11 weeks (Figure 1). The lesion in one male dog never healed, as it was still active after 15 weeks of infection. Some effect of the litter of origin was observed in size and duration of the lesions. Beagles 1 and 6 belonging to one litter had larger lesions, while Beagles 2 and 3 from another litter presented smaller but long-lasting lesions and Beagles 4 and 5 from a third litter had intermediate size lesions. Daily rectal temperature measurements showed weekly increases >38.6°C in all dogs. Pyrexia was also observed in two dogs on day 25 post-infection, just after ulcer formation, reaching 39.3 and 40°C, respectively.

### Pathology and histopathology

**Figure 2:**
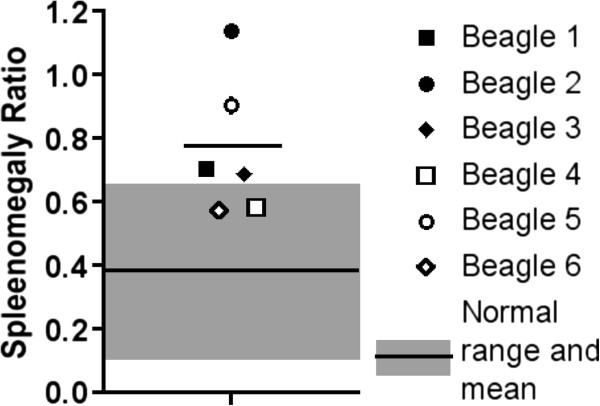
**Splenomegaly ratio of** ***L. (L.) mexicana*** **infected Beagle dogs.** Animals were euthanized at 13-16 months old, 16 weeks after infection. The grey area with the horizontal line represents the mean and standard deviation distribution of normal Beagle dogs of the same age YANG, 1989.

**Figure 3:**
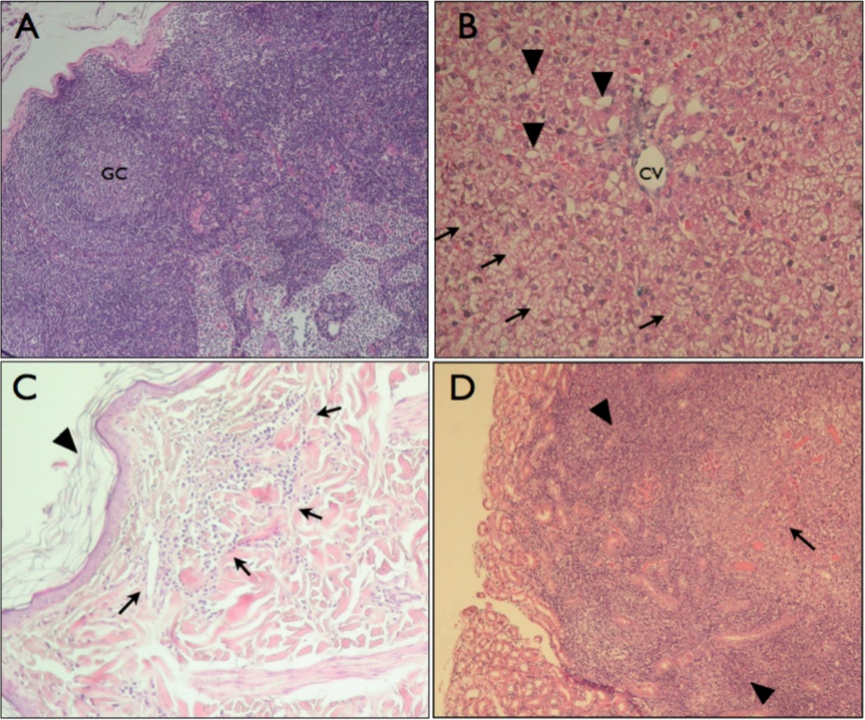
**Photomicrographs of hematoxylin eosin stained sections of tissues from infected dogs. (A)** Cortical zone of the prescapular lymph node. GC: Germinal Centre. Magnification 10×. **(B)** Liver section, magnified 20×. Hepatocytes present hydropic degeneration (arrowhead). Necrotic cells are scattered throughout parenchyma (arrows). CV: Central vein. **(C)** Epidermis section magnified 20×. Inflammatory infiltrate is present in the dermis (arrows) and epidermis integrity is lost by orthokeratotic hyperkeratosis (arrowheads). **(D)** Kidney section magnified 10×. Severe inflammatory infiltrate is observed in the interstitium (arrowheads). Severe lymphocytic and histiocytic inflammatory infiltrate is around necrotic zone (arrow).

Infected animals were euthanized at week 15 post-infection, when lesions had healed except in one dog, and necropsy was carried out to detect any gross abnormality. All analyzed tissues appeared macroscopically normal. Spleen was weighted and the data normalized to body weight. Four of the six dogs (three males and one female) presented a splenomegaly outside the normal range (Figure 2).Biopsies from different tissues were also analyzed for histopathologic damage. Skin tissue from the healed lesions showed focal inflammatory cell infiltration in the dermis around adipose tissue, and a loss of epidermis integrity (Figure 3C). Prescapular lymph nodes presented disseminated atrophy with interstitial edema and an enlarged cortical layer with necrotic fibrinoid zones. There was also an intense vasculitis in this tissue (Figure 3A). The liver of Beagle 4 and 6 showed hydropic and lipid severe degeneration around the central area and multiple focus of oncotic necrosis (Figure 3B). In the kidneys, a severe case of inflammation and interstitial nephritis was found in Beagle 4. There was an extensive histiocytic and lymphocytic infiltrate displacing renal tubules (Figure 3D). Some inflammation was observed in the kidneys of the other infected animals. However, no parasites or infected cells were observed in any of the tissue sections.

### Parasite burden

**Figure 4:**
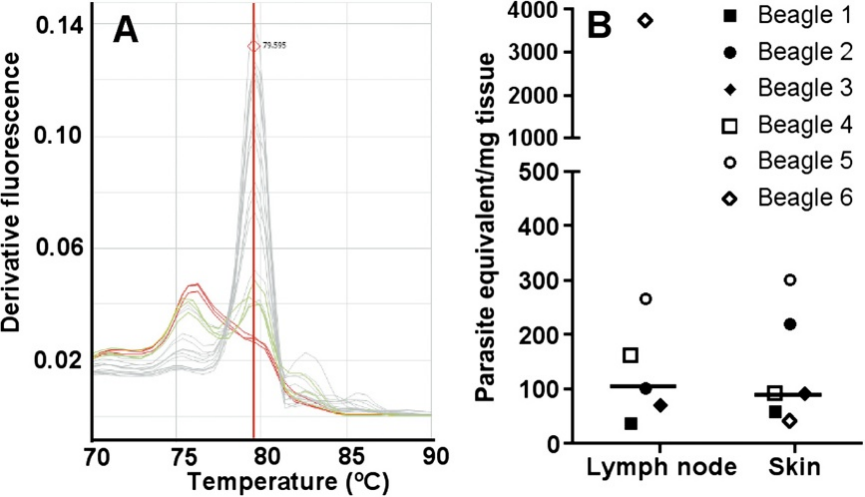
**Quantification of parasite burden by qPCR. (A)** High resolution melting curve of qPCR products showing the specificity of the positive amplification products (gray lines) compared to uninfected negative controls (red curves). **(B)** Parasite burden in the lymph nodes and skin healed lesions at 15 weeks post-infection.

Parasite burden was measured at the site of the healed lesion and in the prescapular lymph node by qPCR targeting *L. (L.) mexicana* kDNA. Parasites were detected in all biopsies from all infected animals, with similar parasite burdens in the skin and in the lymph nodes (Figure 4). Parasite burden varied from 36-3750 parasites equivalent/mg in the lymph nodes and between 57-301 parasites equivalent/mg in the healed lesions. A higher parasite burden in the healed lesion tended to be associated with increased splenomegaly, although this did not reach statistical significance (R^2^ = 0.62, *P* = 0.06), and no association was found with the parasite burden in the lymph nodes.

### Immune response

**Figure 5:**
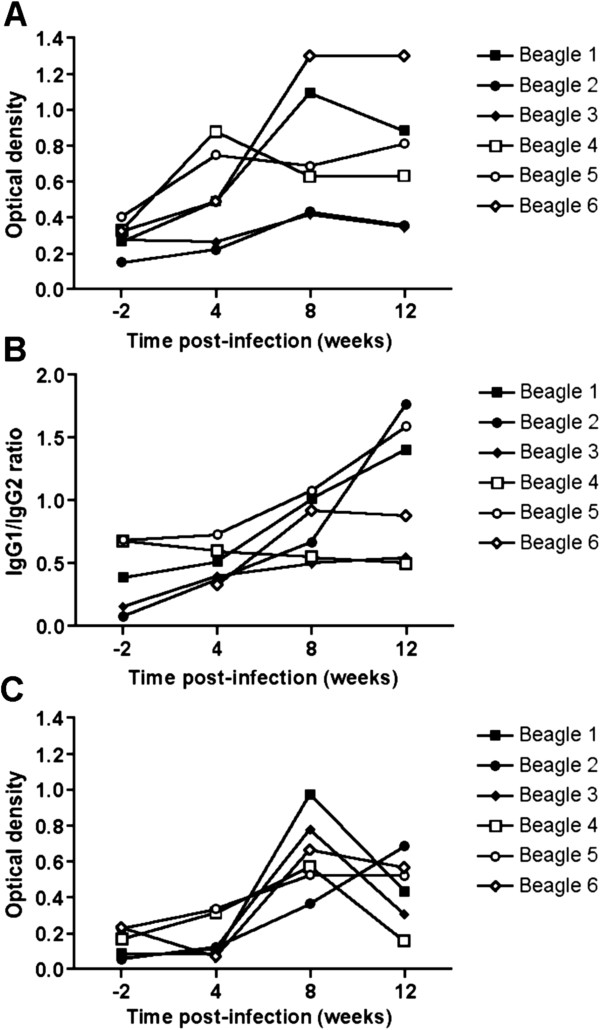
**Antigen** ***-*** **specific humoral immune response in Beagle dogs following infection with** ***L. (L.) mexicana*** **. (A)** Anti-*L. mexicana* total IgG levels were measured by ELISA and read at 415 nm. **(B)** IgG1/IgG2 ratio was similarly measured at 490 nm. **(C)** rNH36-specific IgG levels were determines as anti-*Leishmania* total IgGs.

The immune response of the infected dogs was analyzed to determine whether potential immune responses correlated with disease progression. We measured serum antibody levels as well as IFNγ and IL-10 cytokine levels in serum and tissues at different time points. Anti-*Leishmania* total IgG rapidly increased following infection to reach a maximum level at 8 weeks post-infection. Antibody levels were maintained up to 12 weeks post-infection (Figure 5A). High IgG levels were significantly associated with a large lesion size (R^2^ = 0.87, *P* = 0.005).

Analysis of antibody isotypes showed a gradual IgG isotype switching over the course of infection, from a predominant IgG2 isotype at four weeks post-infection to a predominant IgG1 isotype at 12 weeks post-infection, as indicated by the changes in IgG1/IgG2 ratio (Figure 5B). A high IgG1/IgG2 ratio at 12 weeks post-infection was significantly associated with splenomegaly (R^2^ = 0.68, *P* = 0.04), and tended to be associated with an increased parasite burden in the lesion (R^2^ = 0.42, *P* = 0.15). We also evaluated NH36-specific antibodies. Anti-NH36 IgG levels reached their maximum at 8 weeks post-infection, but then presented some decrease at 12 weeks post infection (Figure 5C).

**Figure 6:**
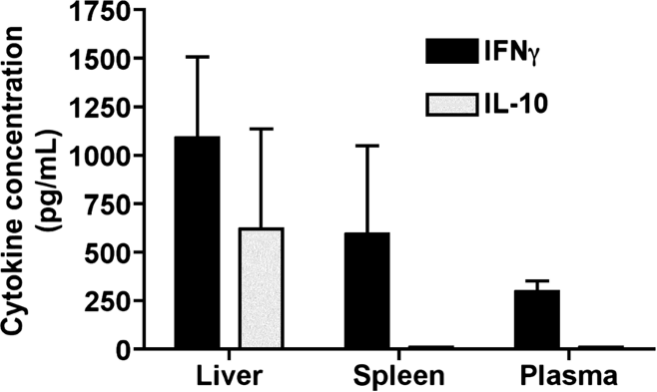
**IFNγ and IL-10 cytokines levels in infected Beagle dogs.** Cytokine concentrations were determined by ELISA in the supernatant of liver and spleen extracts, and in plasma samples. Data are presented as mean and standard deviation.

IFNγ and IL-10 cytokines were measured in liver and spleen tissue homogenates, as well as in serum samples at 15 weeks post-infection, by ELISA. IL-10 could not be detected in spleen or plasma samples, and it was only detected in liver samples (Figure 6). On the other hand, IFNγ was found in all samples and appeared to be the predominant cytokine compared to IL-10 (Figure 6). No significant associations were found between cytokine levels and the other immune or parasitological parameters (*P* > 0.05), although higher IFNγ levels in the spleen and in the liver tended to be associated with lower anti-*Leishmania* IgG, as well as a shorter duration and smaller size of the skin lesions.

## Discussion

While visceral leishmaniasis has been extensively studied in dogs and several experimental models have been described [20, 26, 34, 41–43], very little is known about canine cutaneous leishmaniasis. We present here a first detailed model of experimental infection with *L. (L.) mexicana* in Beagle dogs.

Infection of dogs with *L. (L.) mexicana* parasites led to the development of a typical ulcerated skin lesion in all six infected animals, with a rather rapid onset after 3-4 weeks of infection. Thus, in spite of some variability in terms of lesion size and duration, this infection model appeared very reproducible. A previous attempt of infection of mongrel dogs with 3 × 10^7^ *L. (V.) braziliensis* promastigotes resulted in cutaneous lesion development in only three of four animals, and after a longer and variable incubation period of 8-32 weeks [33]. Lesion duration in the *L. (V.) braziliensis* model was also highly variable, ranging from 16 to 32 weeks [33]. This elevated variability and time length made this former model of limited practical use. A higher parasite dose (1 × 10^9^) has also been used in a model of canine visceral leshmaniasis, but it led to limited clinical signs [44].

The clinical and pathological evolution of *L. (L.) mexicana* we described in this study was also very similar to what has been described for naturally-occurring cutaneous leishmaniasis in dogs. Thus, dogs naturally infected with *L. (V.) braziliensis* or *L. panamensis* present skin lesion varying in size from 0.4-10 cm [27, 29]. Similarly, the histopathologic damage that we observed in the skin and other tissues is very similar to that observed in naturally infected animals [32, 45]. Remarkably, our observation of lymphoid infiltration in the kidney is a first report indicating possible renal damage in experimental cutaneous leishmaniasis. The induction of glomerulonephritis during infection with *L. (L.) infantum* has been recently attributed to the deposition of immune complex following a limited cellular immunity [46]. In addition, it is important to note that *L. (L.) mexicana* can cause a rather wide spectrum of clinical disease, including diffuse and visceral forms in some cases, particularly in patients with low immune status [47–49].

Also similar to the natural infection is the apparent absence of parasites in the healed lesion as assessed microscopically in tissue sections [32]. However, using a technique with higher sensitivity such as qPCR, we clearly demonstrated an important parasite burden up to 15 weeks after infection, both in the skin and in the secondary lymphoid organs of all six infected animals. While this assay relies on the detection of parasite DNA, the presence of live parasites is highly likely, as observed in many animal models of *Leishmania* infection.

These important results suggest that infected dogs have reached an asymptomatic stage, but parasite persistence indicated that, on one hand infection may be reactivated upon alteration of their immune status, and on the other hand, they remain a potential source of infection and may serve as a reservoir of the parasite. In Brazil, it has been clearly established that domestic dogs are the main reservoir of *L. chagasi* [20, 21, 50], but the role of dogs in the transmission of *Leishmania* species causing cutaneous forms of the disease is not well understood [51]. Observations of long lasting ulcer lesions and a high susceptibility to infection in dogs from Argentina suggested that they could play an important epidemiological role in the transmission of *Leishmania* parasites species causing cutaneous leishmaniasis [51]. This has important implications for the definition of epidemiological control measures. Indeed, the massive euthanasia of dogs in Brazil has had a questionable efficacy to reduce infection rate in humans [17], and as a consequence, the World Health Organization has now prioritized the development of veterinary vaccines [34, 41]. Therefore, our model of canine cutaneous leishmaniasis may be very helpful for the evaluation of vaccine candidates. Nonetheless, further development of this animal model may include the evaluation of the addition of sand fly saliva and/or infection by sand fly bites, which have been shown to dramatically alter host immune response to *Leishmania* infection in mice [52], even though its effects in dogs have been less straightforward [41, 42].

Analysis of the immune response of *L. (L.) mexicana* infected dogs indicated an increase in anti-parasite IgG and anti-NH36 IgG levels eight weeks post-infection, which seems much faster than that observed during infection with *L. (L.) chagasi/infantum* in which case antibody levels took more than a year to increase [42, 50]. However, IgG levels appeared to rise faster when intradermal infection was used [53]. Nonetheless, similar to the visceral form of the disease, for which higher IgG levels are associated with symptomatic disease [21], we found that a stronger humoral response was associated with larger lesion size. Interestingly, the analysis of IgG isotypes revealed a gradual shift from a predominant IgG2 to a predominant IgG1 isotype over the course of infection, suggesting an initial Th1 type immune response at the beginning on the infection, followed by progression to a Th2 type response at later stages. This change in immune profile would explain the initial control of the lesion leading to its healing, as well as the failure of the immune system to achieve parasite elimination, allowing the detection of *Leishmania* parasite DNA in the healed lesion and lymph nodes. It is also in agreement with the pathogenic role reported for IgG1 with *L. (L.) mexicana* infection in murine models [54]. However, cytokine measurements showed a significant IFNγ production in plasma, liver and spleen, while IL-10 was only detected in the liver. A rather mixed Th1/Th2 response is thus more likely to be occurring, as reported in canine visceral leishmaniasis, for which a clear immune polarization has been difficult to observe [43, 55–62]. Nonetheless, a Th1 immune response characterized by predominant IgG2 antibodies and higher IFNγ production seem rather associated with parasite control, as evidenced by a significant association with a reduced splenomegaly, and a tendency to lead to a decreased lesion size and duration. This is in agreement with established mouse models [63], as well as observations on canine visceral leishmaniasis. The analysis of additional cytokines and tissues, such as draining lymph nodes, should help further characterize the immune response in this animal model.

## Conclusions

We present here the first model of canine cutaneous leishmaniasis caused by *L. (L.) mexicana*. This reproducible model presented typical ulcerated skin lesions in all animals, with characteristics very similar to those observed in naturally infected animals. Parasitological analysis clearly showed the long-term persistence of parasite both in healed skin lesions and the lymph nodes. Also, infection was associated with a mixed Th1/Th2 response, although a Th1 profile seemed to be associated with better parasite control. This model will be very useful to further clarify the role of dogs as reservoirs and in *L. (L.) mexicana* transmission cycles. Further studies should also provide new insights on the immunopathogenesis of canine cutaneous leishmaniasis. Finally, this model will be very useful for the evaluation of novel vaccine and drug candidates.
